# Safety and Efficacy of Autologous Bone Marrow Derived Mononuclear Cell Transplant in the Management of Various Neurological Disorders

**DOI:** 10.7759/cureus.75617

**Published:** 2024-12-12

**Authors:** Sanjay Kala, Anchal Aggarwal, Bhagat Singh Rajput, Chayanika Kala, Santosh K Barman

**Affiliations:** 1 Department of General Surgery, GSVM Medical College, Kanpur, IND; 2 Department of Regenerative Medicine and Cell Based Therapies, GSVM Medical College, Kanpur, IND; 3 Department of Pathology, GSVM Medical College, Kanpur, IND; 4 Department of Community Medicine, GSVM Medical College, Kanpur, IND

**Keywords:** bone marrow-derived mononuclear cells, functional improvement, neurological disorders, quality of life, regenerative medicine

## Abstract

Background: Cerebral palsy (CP), traumatic spinal cord injury (SCI), and muscular dystrophy (MD), among the various other neurological disorders, are major global health problems because they are chronic disorders with no curative treatments at present. Current interventions aim to relieve symptoms alone and therefore emphasize the necessity for new approaches.

Objective: This study aims to assess the safety and efficacy of autologous bone marrow-derived mononuclear cell (BM-MNC) therapy in patients with CP, traumatic SCI, and MD. Functional improvement and safety are the primary outcomes, while secondary outcomes include patient-reported improvement in quality of life.

Methods: This was a single-arm, open-label prospective study conducted on 100 patients with CP, SCI, and MD at the GSVM Medical College, Kanpur, India. Bone marrow aspirates were processed via centrifugation, and autologous BM-MNCs were administered intrathecally or intramuscularly. Gross motor function classification system (GMFCS) for CP, the American spinal injury association (ASIA) motor score for SCI, and the north star assessment for limb girdle type dystrophies (NSAD) for MD were used for functional outcome assessment at baseline and at post-treatment cycles. Informed consent was obtained, and the study was approved by the ethics committee. Paired t-tests were used to analyze statistical significance (p<0.05).

Results: Functional improvements were significant with autologous BM-MNC therapy. Improved motor and cognitive function were shown in CP patients with reduced GMFCS scores (p<0.001). Upper and lower extremity ASIA motor scores improved markedly (p<0.001) in SCI patients. Stabilized muscle strength was seen in MD patients, with increased NSAD and activities of daily living (ADL) scores, suggesting slowing of the disease progression (p<0.019). Side effects were mild and transient.

Conclusion: Autologous BM-MNC therapy appears to be a promising, minimally invasive option for patients with CP, SCI, and MD, which appears to markedly improve functional outcomes and quality of life and may therefore be relevant to clinical practice.

## Introduction

Cerebral palsy (CP), traumatic spinal cord injury (SCI), and muscular dystrophy (MD) are major neurological disorders that are a global health challenge, resulting in long-term disability, reduced life quality, and substantial economic and social burden due to their chronic and progressive nature. Because these disorders are chronic, with some of them being progressive with no curative treatments, patients, caregivers, and healthcare systems are burdened. CP is a motor disorder caused by early brain damage and can result in varying degrees of physical and cognitive impairments that impair mobility and quality of life. SCI is caused by trauma and affects spinal cord function leading to loss of motor and sensory abilities. MD is a group of genetic disorders that cause progressive muscle wasting, resulting in severe physical disabilities and frequently premature mortality [1,2]. These disorders are collectively significant contributors to disability-adjusted life years (DALYs) and contribute to high socioeconomic costs through long-term care needs, rehabilitation, and ongoing supportive interventions [3,4].

Current therapies are generally limited in success, consisting primarily of palliative and symptom-focused treatments, highlighting the need for novel therapies that could potentially restore lost functions or slow further degeneration. Over the past years, regenerative medicine has come forward as a promising field to address these unmet needs, with cell-based therapies offering the possibility of directly targeting and healing damaged tissues. In the cell-based therapies under investigation, bone marrow-derived mononuclear cell (BM-MNC) therapy has shown promise for its regenerative capabilities in neurological and neuromuscular disorders. Autologous BM-MNC therapy harnesses a wide range of cells in a diverse mix of mesenchymal stromal cells and hematopoietic stem cells, each with distinct immunomodulatory, anti-inflammatory, and regenerative properties. The capacity of these cells to release trophic factors that support tissue repair, inhibit apoptosis, and foster neuroprotection is important for treating conditions with limited benefit from traditional treatments [5,6].

Autologous BM-MNCs can migrate to injury sites and exert therapeutic effects by neuroprotective and angiogenic mechanisms. It has shown promise in promoting axonal sprouting and remyelination and improving motor and sensory functions in SCI animal models. Studies in CP show functional and metabolic improvement associated with autologous BM-MNC neurogenesis and neuroprotection, which may contribute additional benefits beyond current therapies. As in MD, autologous BM-MNC therapy is promising for the repair and stabilization of disease progression, as studies suggest that autologous BM-MNC therapy can sustain muscle function over the long term [7,8]. Additionally, because autologous BM-MNCs are obtained from the patient’s bone marrow, the therapy reduces the risk of immune rejection, which is an important advantage over other cell-based treatments [9].

This study evaluates the therapeutic potential of autologous BM-MNC therapy in improving functional outcomes and quality of life in CP, SCI, and MD patients. The details of autologous BM-MNC preparation and administration, including intrathecal and intramuscular delivery, depending on the patient’s condition, are covered. In this study, standardized tools that quantify motor and sensory improvements were used for the evaluation of functional outcomes, including the gross motor function classification system (GMFCS) for CP, the American spinal injury association (ASIA) motor score for SCI, and the north star assessment for limb girdle type dystrophies (NSAD) for MD. The documentation of adverse events is closely monitored for safety, and patient-reported improvement in quality of life is used as a secondary outcome measure to help assess the impact of therapy on overall well-being [10].

The goal of this study is, therefore, to systematically evaluate the safety, efficacy, and other important features of autologous BM-MNC therapy in patients with CP, SCI, and MD. Specific objectives are to quantify improvements in functional measures within motor domain and cognitive domains, as well as patient quality of life, and to establish a detailed safety profile from adverse event data. This research aims to provide clinically meaningful insights into the potential of autologous BM-MNC therapy as a regenerative option, to fill current gaps in treatment, and to expand therapeutic options for patients with chronic debilitating neurological disorders.

## Materials and methods

Study design

A single-arm open-label prospective clinical study was done in the Department of General Surgery, GSVM Medical College, Kanpur. The study was conducted from May 2023 to August 2024, and approval from the institute's ethics committee was obtained on 23rd April 2023. This study included 100 patients with neurological disorders, including CP, traumatic SCI, and MD, with a distribution of 40 cerebral palsy (CP), 50 spinal cord injury (SCI), and 10 muscular dystrophy (MD). Patients who met certain inclusion criteria were recruited, and their clinical outcomes were subsequently monitored after autologous BM-MNC therapy.

The sample size for this study was calculated with a confidence level of 95% and a statistical power of 80%. The calculation was based on an anticipated mean functional improvement of 20%, with a standard deviation of 10%. Using conventional sample size determination formulas, the minimum required sample size was estimated to be 88%. To enhance reliability and account for potential dropouts, the study enrolled a total of 100 patients. This ensured adequate representation across the three neurological disorders and maintained sufficient statistical power to detect meaningful outcomes.

Ethical details

This study is reviewed and approved by the Institutional Ethics Committee of GSVM Medical College, India, following the guidelines of the Indian Council of Medical Research (ICMR) and according to the Council of New Drugs and Clinical Trial Rules, 2019. The ethical principles conform to the World Medical Association's Declaration of Helsinki on conducting research with human subjects. The meeting of the Ethics Committee (For Biomedical Health and Research), GSVM Medical College, Kanpur, was held on 13th April 2023 at GSVM Medical College, Kanpur, and the approval was obtained on 23rd April 2023 under reference number EC/BMHR/2023/10. Informed written consent was obtained from the adult patients, while parents’ consent was sought for the children. The study has also been registered with the Clinical Trials Registry India (CTRI), with the registration details CTRI/2024/10/074805.

Inclusion and exclusion criteria

**Figure 1:**
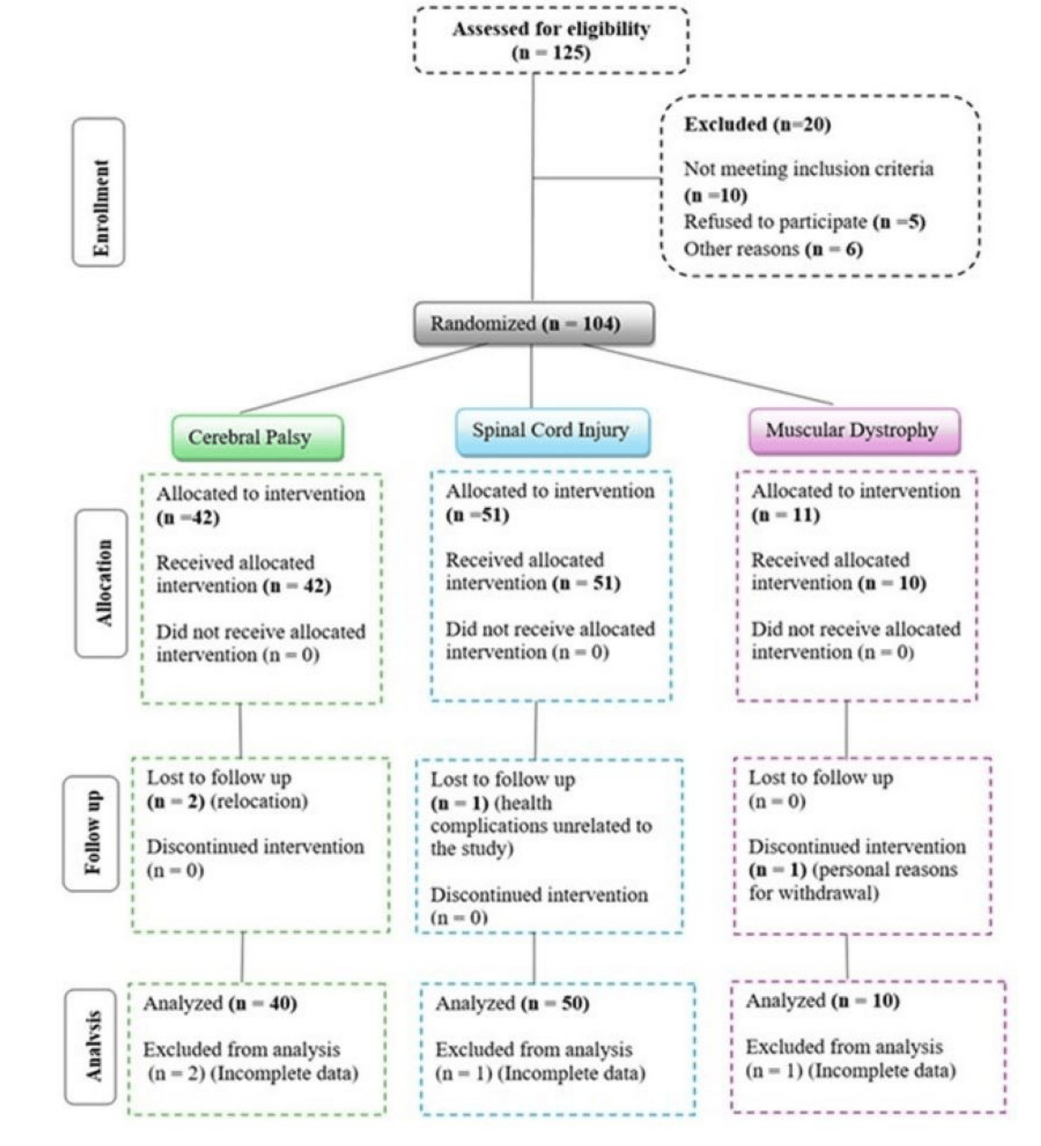
CONSORT flow diagram for the search methodology.

Patients aged 1 to 65 years with a diagnosis of traumatic SCI, CP, or MD with no effective standard treatment options are included as inclusion criteria for this study (The CP group consisted of children aged 1-18 years with varying levels of motor dysfunction, the SCI group included both adolescents (ages 13-18) and adults (ages 19-65) who had experienced traumatic spinal cord injuries, and the MD group included patients aged 1-65 years diagnosed with muscular dystrophy, at different stages of disease progression). Participants must also commit to attending regular follow-up sessions per the study protocol. Exclusion criteria comprise patients with organic brain diseases, central nervous system infections, severe systemic conditions (such as HIV, hepatitis, or malignancy), active autoimmune disorders, known allergies to autologous BM-MNC therapy, non-compliance with study requirements, or inability to provide informed consent. Figure 1 shows the search methodology.

Sample collection and autologous BM-MNC preparation

A bone marrow sample was taken aseptically; it was mostly taken from the posterior superior iliac crest. In the adults, 80-100 ml of the marrow was aspirated (40-50 ml in children) with the help of a heparinized Jamshedi needle and filtered using a blood transfusion set to get 50 to 60 ml filtered BM, which was transferred into two Tubex BMC tubes (25 ml each). Post centrifugation-step 1 (3500 RPM X 6 minutes) and step 2 (3300 RPM X 5 minutes), 6 ml of concentrate was obtained from each processing tube, of which the top 4 ml concentrate and the remaining bottom 2 ml were collected separately. The mononuclear cell layer ( bottom 2ml concentrate ) was then harvested, counted for viability using trypan blue dye, and enumerated for CD34+ cells by FACS. The average viability was 98 percent. The autologous BM-MNCs were then suspended for intrathecal or intramuscular administration. The patients with spinal cord injuries (SCI) were primarily injected with intrathecal injections containing about 20 million cells per mL to target the central nervous system. Patients with Cerebral Palsy (CP) and Muscular Dystrophy (MD) received intramuscular injections with the same cell concentration to improve motor function and muscle strength.

Neurorehabilitation protocol

After autologous BM-MNC transplantation, all patients commenced neurorehabilitation in the form of physiotherapy, occupational therapy, and speech therapy. Rehabilitation was intended to provide functional gains and maximize the therapeutic utility of functional activities in motor, cognitive, and quality-of-life domains. Patients remained with neurorehabilitation for the entire period of the follow-up.

Outcome measures

The primary outcomes assessed the safety profile of autologous BM-MNC therapy by monitoring the incidence of adverse events post-transplant and evaluating functional improvements using disorder-specific scales. For CP, the GMFCS and MOCA scales were used; for SCT, the ASIA scale and MMT scores; and for MD, the NSAD and MMT scores were used. Secondary outcomes focused on patient-reported quality of life improvements, measured through the ADL score. These measures allowed for tracking motor function, sensation, and quality of life effectively.

Statistical analysis

Data was collected and analyzed using Statistical Package for Social Sciences (SPSS software version 26) [11]. Simple descriptive statistics were used to analyze priorities, with paired t-tests being used in the analysis of associations of variables with significance at p<0.05. Data was summarized to show the effectiveness indicators and the changes that occurred in the course of the study.

## Results

Patient demographics

The study cohort consisted of 100 patients diagnosed with neurological disorders. Among these, 40 patients had CP, 50 had traumatic SCI, and 10 had MD. The mean age was 9 years for CP patients, 31 years for those with SCI, and 11 years for those with MD. The overall gender distribution across the cohort was 79% male and 21% female (Table 1).

**Table 1:** Demographics and baseline characteristics.

Disorder	Number of patients	Mean age (years)	Gender distribution (M/F)
Cerebral palsy	40	9	25/15
Spinal cord injury	50	31	45/5
Muscular dystrophy	10	11	9/1

Cerebral palsy

The patients with CP showed significant changes in functional parameters after autologous BM-MNC therapy. The GMFCS scores were significantly enhanced, with a change from the baseline mean of 40% to as low as 15% after three cycles of therapy (p<0.001). The muscle strength and MoCA scores were significantly better in the intervention group with p<0.001. Significant improvements were also noted in sitting balance, standing balance, and walking balance, and in walking ability (p<0.001).

**Figure 2:**
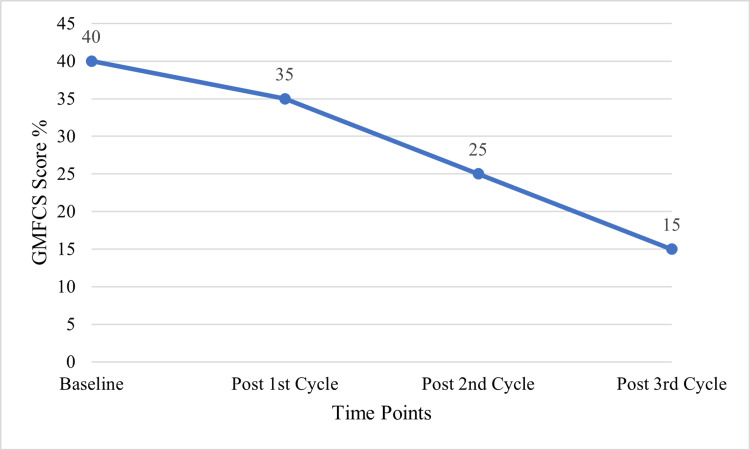
GMFCS score progression in cerebral palsy.

Figure 2 illustrates the steady decline in GMFCS scores across four assessment points: Baseline (Pre-therapy), Post 1st Cycle, Post 2nd Cycle, and Post 3rd Cycle. The scores started at 40%, then dropped to 35%, 25%, and finally 15% by the third cycle, which corresponded to cumulative improvements in motor function with autologous BM-MNC therapy. These results support the idea that BM MNC therapy may be an effective means of improving motor function in CP patients.

Traumatic spinal cord injury

In traumatic SCI patients, Autologous BM-MNC therapy helped achieve a major improvement in motor and sensory areas. The ASIA motor scores showed significant improvement after the therapy. Motor scores of the upper extremities also increased on the right and left sides, from 33.04±12 at baseline to 40.8±7.3 after three cycles (p<0.001). Likewise, the lower extremity motor scores increased from the baseline mean of 19.2±9.4 to 35.1±6.4 after three cycles (p<0.001). Sensory function, assessed by light touch and pinprick scores, was also improved significantly.

**Figure 3:**
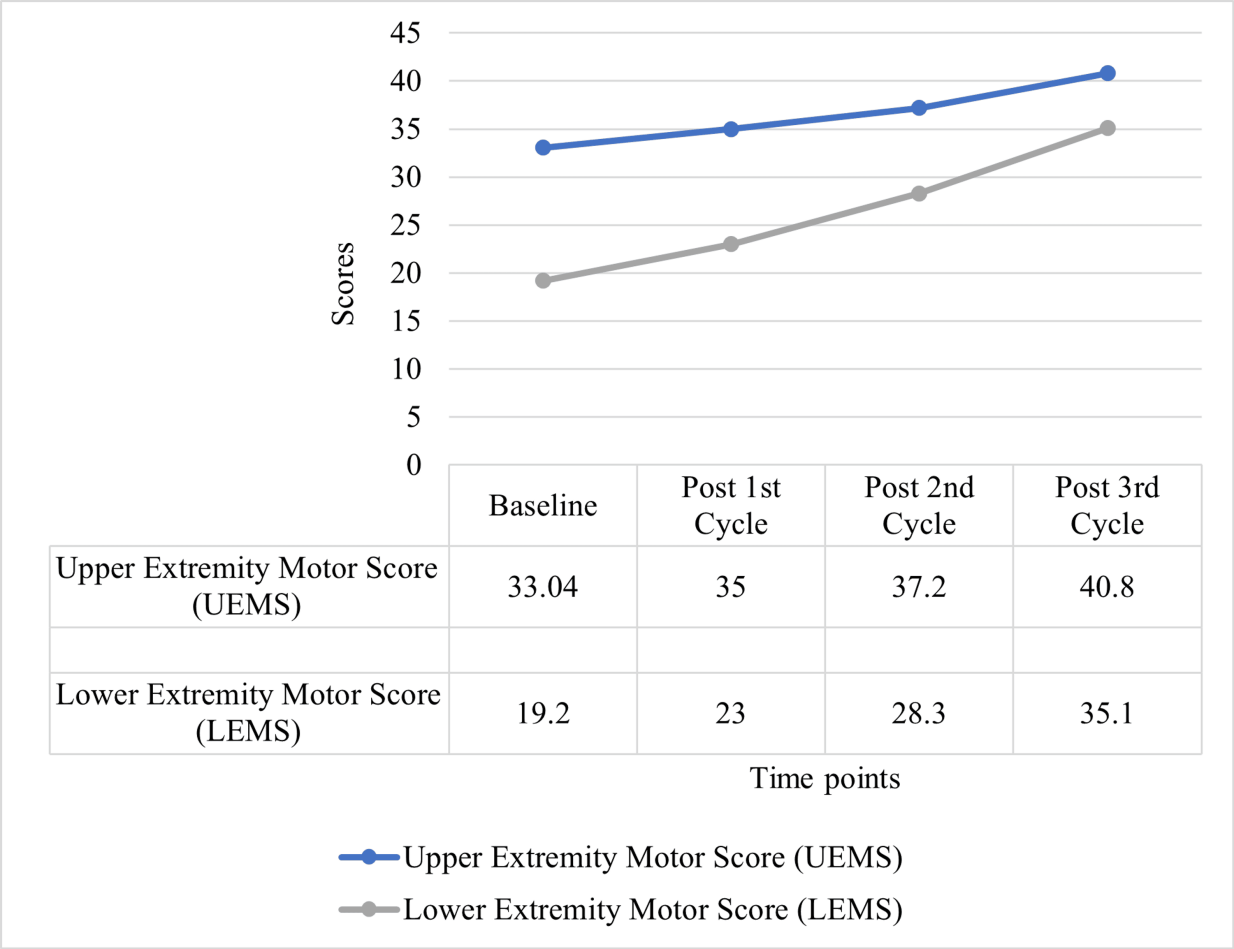
Motor-score progression in spinal cord injury patients.

Figure 3 displays the progression of motor scores in traumatic SCI patients, specifically showing improvements in upper extremity motor score (UEMS) and lower extremity motor score (LEMS) across four time points: Baseline (Pre-therapy), Post 1st Cycle, Post 2nd Cycle, and Post 3rd Cycle. At baseline, UEMS is 33.04, and LEMS is 19.2. After the first cycle of autologous BM-MNC therapy, both scores improve; UEMS is 35, and LEMS is 23. These improvements persist in the subsequent treatment cycles; UEMS scores were 37.2 and 40.8, while LEMS scores were 28.3 and 35.1 after the second and third cycles, respectively. The progress in UEMS and LEMS has proven steady, and, more importantly, there has been a significant improvement in motor function of the upper and lower limbs in the course of the therapy. This implies that autologous BM-MNC therapy may promote functional recovery in patients with traumatic SCI and, therefore, improve strength and motor function. The gradual improvement of the motor scores over the treatment cycles also suggests an additive effect of the therapy and supports the use of the therapy as a potentially helpful treatment for patients with spinal cord injuries.

Muscular dystrophy

The MD patients also had positive responses to autologous BM-MNC therapy, as evidenced by the enhanced NSAD, MMT, and ADL scores. NSAD scores were higher at baseline, 12.8±7.9, and after the third cycle, 14.8±8.8 (p<0.019), suggesting better muscular function. On the right side of MMT, the score improved from 35.8±7.8 to 37.3±8.3, and on the left side, from 36.5±7.3 to 37.6±7.5 (p<0.010). ADL was also significantly improved from 6.5±2.9 at baseline to 5.6±2.9 (p<0.003).

**Figure 4:**
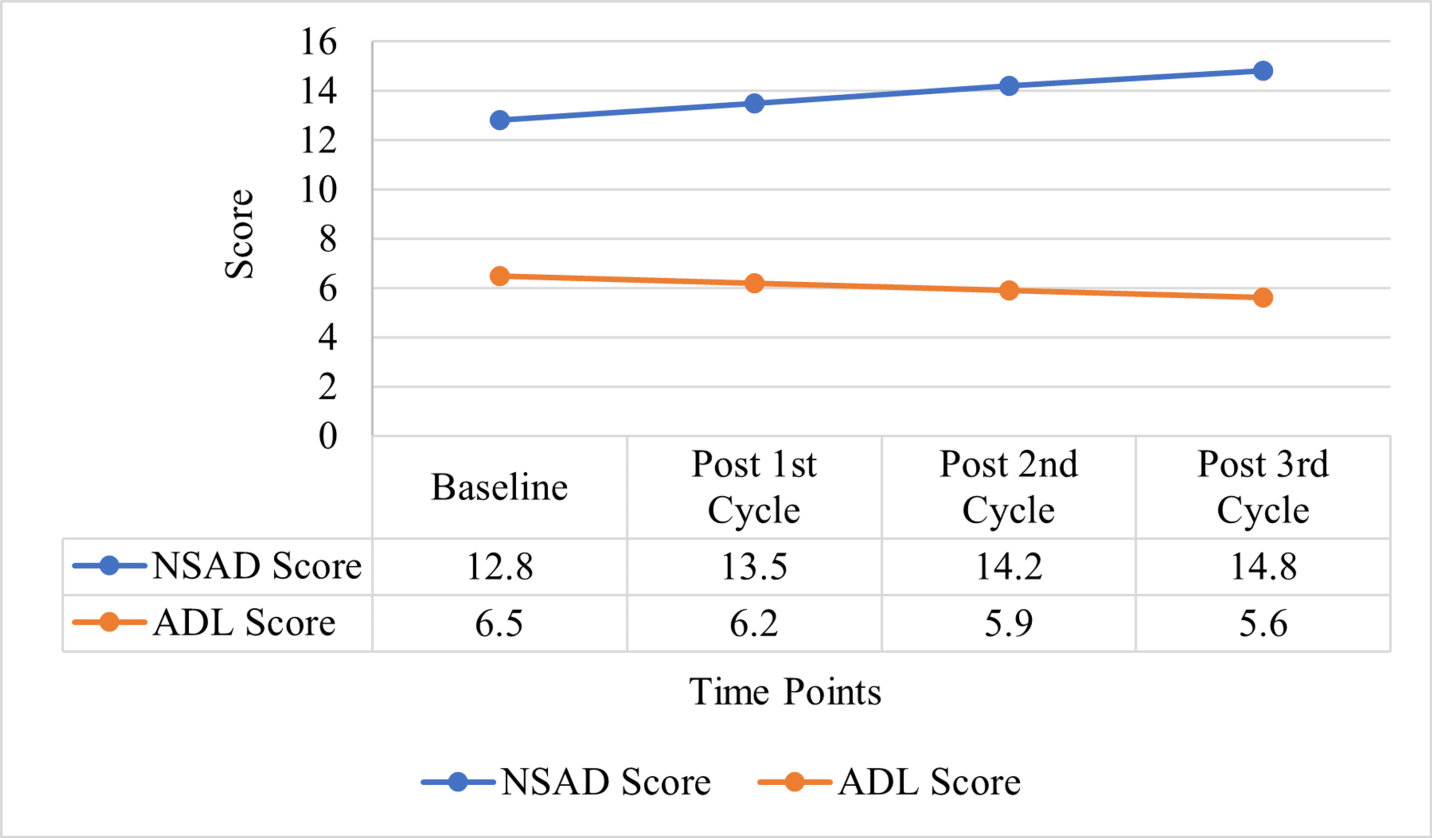
Score progression in muscular dystrophy patients (NSAD and ADL). NSAD: north star assessment for limb girdle type dystrophies; ADL: activities of daily living.

Figure 4 shows the improvement of the scores in muscular dystrophy patients for NSAD and ADL scores over the different cycles of treatment is depicted in figure 3. Mobility and physical activity after autologous BM-MNC therapy, in relation to the treatment approach. Figure 3 illustrates the progression of NSAD and ADL scores in MD patients across four time points: Baseline (Pre therapy), Post 1st Cycle, Post 2nd Cycle, and Post 3rd Cycle. At baseline, the NSAD score is 12.8 and the ADL score is 6.5. After the first cycle of autologous BM-MNC therapy, the NSAD score is 13.5, and the ADL score is 6.2. This trend progresses to the next cycles, where NSAD scores rise to 14.2 and 14.8 in the second and third cycles, respectively, with decreasing ADL scores of 5.9 and 5.6. A progressive step-wise rise in NSAD scores denotes improved muscle strength, and the higher NSAD is a sign of better limb function. On the other hand, the decreasing ADL scores are indicative of better functional status in terms of daily living activities because lower ADL scores are preferred. Altogether, these findings indicate that autologous BM-MNC therapy improves muscle function and daily life quality in MD patients and that the effects accumulate with subsequent cycles of treatment. This data suggests that autologous BM-MNC therapy may be a useful supportive treatment for addressing the functional deterioration in muscular dystrophy (Table 2).

**Table 2:** Summary of key functional scores before and after autologous BM-MNC therapy. SD: standard deviations; GMFCS: gross motor function classification system; MoCA: Montreal cognitive assessment; UEMS: upper extremity motor score; LEMS: lower extremity motor score; NSAD: north star assessment for limb girdle type dystrophies; ADL: activities of daily living.

Disorder	Scale	Baseline (Mean±SD)	Post 3 Cycles (Mean±SD)
Cerebral palsy	GMFCS	40%	15%
	MoCA	23.65±2.4	24.55±2.0
Spinal cord injury	UEMS (Right)	16.92±6.1	20.92±3.5
	LEMS (Right)	9.74±5.0	18.0±3.3
Muscular Dystrophy	NSAD	12.8±7.9	14.8±8.8
	ADL	6.5±2.9	5.6±2.9

Safety

In summary, autologous BM-MNC therapy was safe, and no serious adverse effects were observed in the present study. They also reported some peripheral aversive reactions, such as headache, nausea, vomiting, fever, and backache, which lasted for only one week. In the MD group, five patients complained of headaches, two of which had nausea and pain. Two of the SCI patients had mild fever, and several patients complained of back pain after the procedures. In the CP group, one child complained of severe headache accompanied by nausea and vomiting.

Statistical findings

All the differences between the baseline and post-treatment measurements were statistically significant, with p<0.05. For CP patients, GMFCS, sitting balance, standing balance, and ambulation were significant with p<0.001. Motor scores of the upper and lower extremities, as well as sensory scores for light touch and pinprick, were significantly improved in SCI patients, and the p-values were less than 0.001. In MD patients, both NSAD and ADL scores improved significantly, with p<0.019 and p<0.003, respectively.

## Discussion

This paper shows that autologous BM-MNC therapy holds some level of effectiveness in a broad range of neurological disorders such as CP, SCI, and MD. In CP, patients had better neurological and motor functions, as evidenced by better GMFCS scores, muscle strength, and MoCA scores. Most importantly, the GMFCS scores reduced from 40% at the beginning to 15% after three cycles of treatment, along with the improvements in balance and ambulation, which prove the functional benefits of autologous BM-MNC therapy [12,13].

The therapy proved highly beneficial in traumatic SCI patients in terms of motor and sensory recovery, as reflected by improved scores on the ASIA motor scale as well as enhancement of upper and lower extremity scores post-therapy. This is in line with the general functional enhancement, which supports the possibility of autologous BM-MNC therapy in the recovery of SCI [14,15]. In MD patients, there was an increase in the NSAD and ADL scores and a relatively stable muscle strength over time. The maintenance of muscle strength after therapy is a positive result, especially since muscular dystrophies are progressive diseases [16].

These results are in concordance with other studies that have demonstrated the neurorestorative effects of autologous BM-MNC therapy in neurological diseases. For example, previous research in CP has indicated comparable gains in motor and cognitive development, which indicates that autologous BM-MNCs offer neurotrophic and neurorestorative benefits by releasing growth factors and anti-inflammatory agents [17]. In SCI, previous researches have reported increased axonal regeneration, and enhanced sensory and motor function, and bladder function after autologous BM-MNC treatment. Such effects are explained by the release of trophic factors that promote nerve regeneration and decrease neuroinflammation [18]. In the context of MD, autologous BM-MNC therapy has been identified for its ability to slow down muscular deterioration due to its ability to promote muscle fiber regeneration, which is in concordance with this study where patients with MD had better NSAD and ADL scores [19,20].

The observed therapeutic effects of autologous BM-MNCs can be explained by several factors that affect the development of the disease and promote clinical recovery. Autologous BM-MNCs consist of a diverse cell population, such as hematopoietic and mesenchymal stem cells, that are involved in tissue repair through the secretion of signaling molecules. Many of these cells, for example, release neurotrophic factors, cytokines, and angiogenic factors such as vascular endothelial growth factor (VEGF) and fibroblast growth factor (FGF), which enhance neurogenesis, inhibit inflammation, and prevent apoptosis of neurons. In CP, these factors help in the rewiring of the neural circuits and, consequently, in motor and cognitive rehabilitation. Regarding SCI, autologous BM-MNCs participate in axonal sprouting, remyelination, and the degradation of scar tissue, which restores motor function. In MD, the ability of autologous BM-MNCs to regenerate muscles and replace the damaged fibers is clinically relevant for treating progressive muscle atrophy [21,22].

Subsequent studies should also consider evaluating treatment factors, including the number of cells, route of administration, and the frequency of the therapy cycles. Additional improvements in the quality of the autologous BM-MNCs could be achieved by reducing variability in cell viability and quality caused by the patient’s characteristics. Furthermore, knowledge of the exact processes through which autologous BM-MNCs study may open the door to further targeted therapies, which could lead to the development of individualized treatment plans that would yield the highest levels of patient benefit [23,24].

This study indicates that autologous BM-MNC therapy could be a safe and effective treatment in managing otherwise untreatable neurological disorders such as CP, traumatic SCI, and MD. The therapy shows promise in not only the motor and sensory domains but also in the QoL of the patients. In accordance with the literature pertaining to cell-based therapies. These results show the efficacy of autologous BM-MNCs in providing tissue repair and functional remission in neurodegenerative disorders [25].

The clinical relevance of this study is, therefore, profound since autologous BM-MNC therapy is a minimally invasive, autologous treatment modality for patients with few therapeutic options. The safety profile of the therapy and the potential for regeneration make the therapy valuable in clinical practice for the treatment of neurological disorders. Due to its ability to target various aspects of disease pathogenesis, autologous BM-MNC therapy may be a key therapeutic approach in regenerative medicine for neurodegenerative and neuromuscular diseases.

Limitations and future research

Several limitations that are inherent in the present study should be borne in mind: First, the study had a relatively small number of participants, particularly the MD patients; thus, generalization of results may be a concern. Secondly, the single-arm open-label study design’s inherent bias could affect the efficacy observed, though this does not seem to be the case. Further investigations with respect to this subject should include randomized controlled trials with comparatively greater population sizes and define clear procedures for autologous BM-MNC treatment. However, the safety and effectiveness of the treatment beyond 12 months are not known. This suggests an unmet need for further research, including longer-term studies of autologous BM-MNC therapy to confirm the sustained functional benefits.

## Conclusions

This study shows that autologous BM-MNC therapy is a safe and possibly efficacious treatment for CP, SCI, and MD. The therapy was associated with improvements in functional outcome measures, including the GMFCS in CP, the ASIA motor score in SCI, and the NSAD score in MD, and was well tolerated. The observed functional improvements, such as motor and sensory functions, support the therapeutic value of BM-MNC in meeting the unmet needs of patients with few treatment options. However, the study acknowledges the limitations of a single-arm, open-label design and the need for standardization of adjunct therapies to reduce variability in outcomes. In clinical application, BM-MNC therapy is a minimally invasive, self-mobilizable procedure that eliminates risks like immune rejection and complications. Its use in various neurological disorders also shows its efficacy and could be used as an additional therapy in regenerative medicine. As noted, the effectiveness of the therapy in terms of quality of life and physical outcomes makes it clinically valuable to patients with severe neurological disorders. Further studies should involve many centers, a greater number of patients, and randomized controlled trials to establish the validity of these observations and determine the long-term outcomes and risks. Furthermore, investigations of the most potent cell concentration, dosage, and administration frequency for BM-MNCs would improve their therapeutic efficacy and reproducibility in the clinic.
